# Supplementary material for: Optical imaging of localized chemical events using programmable diamond quantum nanosensors
Source: Nat Commun. 2017 Mar 20;8:14701. doi: 10.1038/ncomms14701 (PMC5364376; doi:10.1038/ncomms14701)
Supplement: Supplementary Information — Supplementary Figures, Supplementary Methods and Supplementary References. [file ncomms14701-s1.pdf]

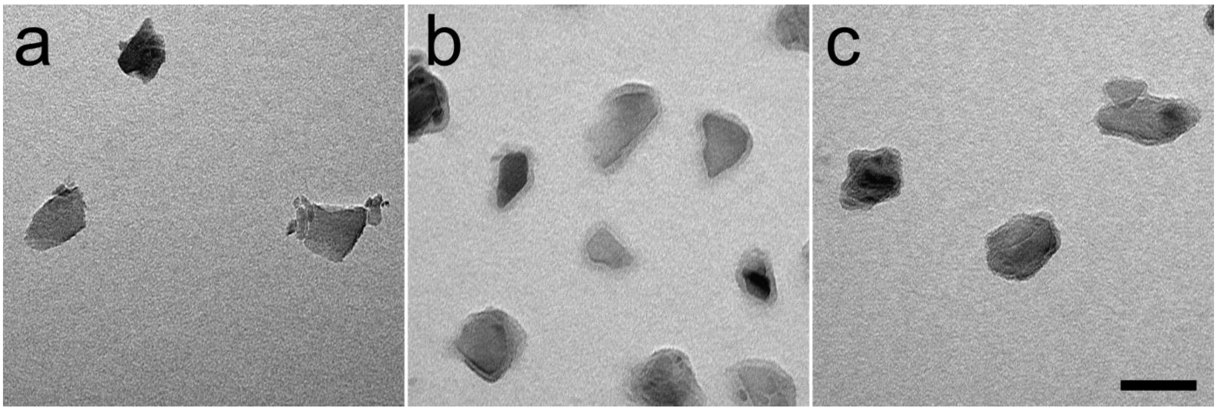

Supplementary Figure 1. Typical TEM image of various ND particles. (**a**) non-coated oxidized NDs. (**b**) Polymer coated ND-HPMA. (**c**) ND@redox. The scale bar represents 50 nm.

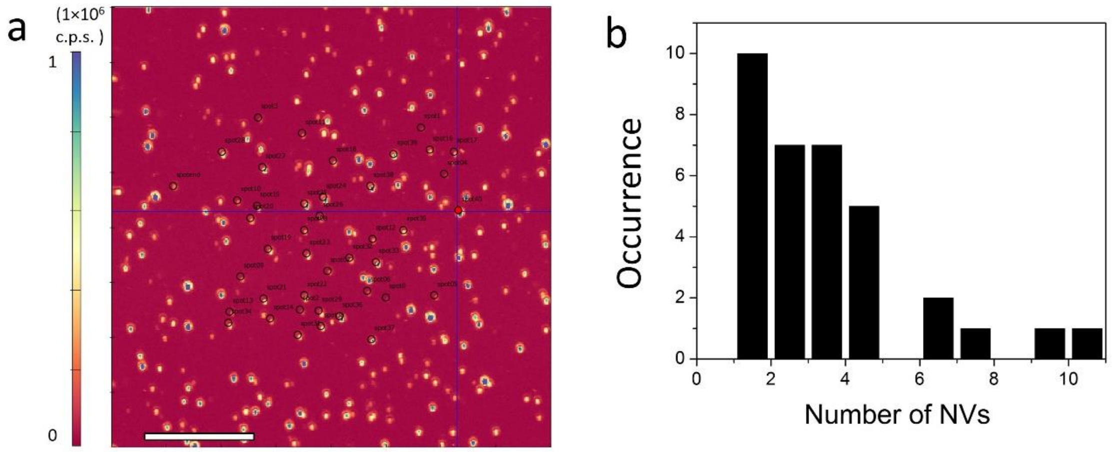

Supplementary Figure 2. Number of NV centers per particle. The number was calculated through analysis of 40 randomly chosen ND particles. (**a**) Typical confocal image of ND-HPMA particles dispersed on top of coverglass. The scale bar represents 10 µm. (**b**) The distribution of the number of NV centers in selected (marked with black circle) spots in (**a**); the number of NV centers in an individual particle was determined from the autocorrelation function $g^{(2)}(\tau)$.

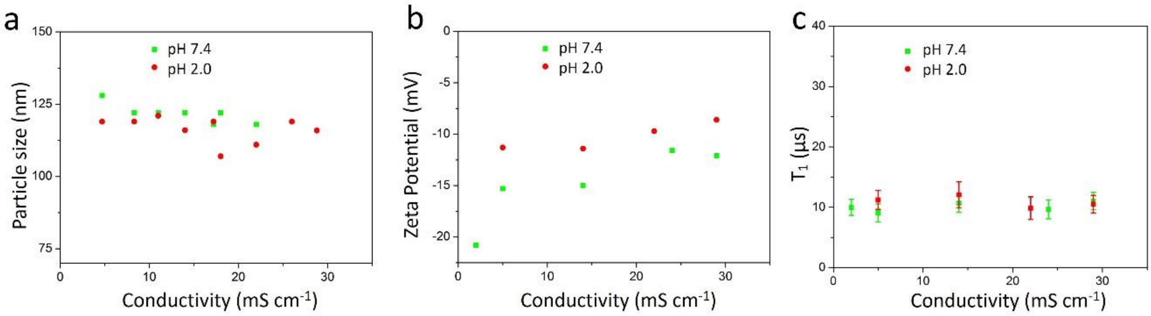

Supplementary Figure 3. The influence of ionic strength on the behavior of ND-HPMA-Gd. (**a**) Hydrodynamic radii, (**b**) zeta potentials, and (**c**) T_1 ensemble measurement of ND-HPMA-Gd in pH 7.4 and pH 2.0 buffers. The ionic strength is expressed as conductivity.

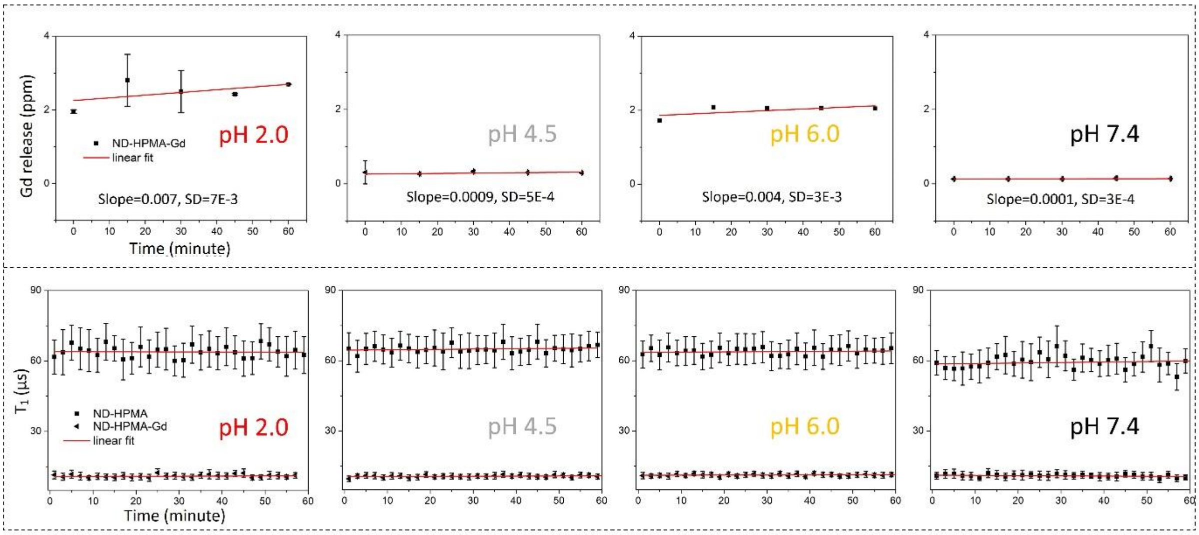

Supplementary Figure 4. Behavior of the control sample in different pH buffers. Release kinetics of Gd^{3+}-complex from ND-HPMA-Gd (non-cleavable) checked by ICP MS (upper panel) in different buffers (pH 2.0, 4.5, 6.0, and 7.4). Time-dependent ensemble measurement for T_1 (lower panel) of ND-HPMA and ND-HPMA-Gd (non-cleavable) in different buffers (pH 2.0, 4.5, 6.0, and 7.4). The slope of all linear fit is approaching zero, indicating negligible change of Gd^{3+} -complex within the measurement time period.

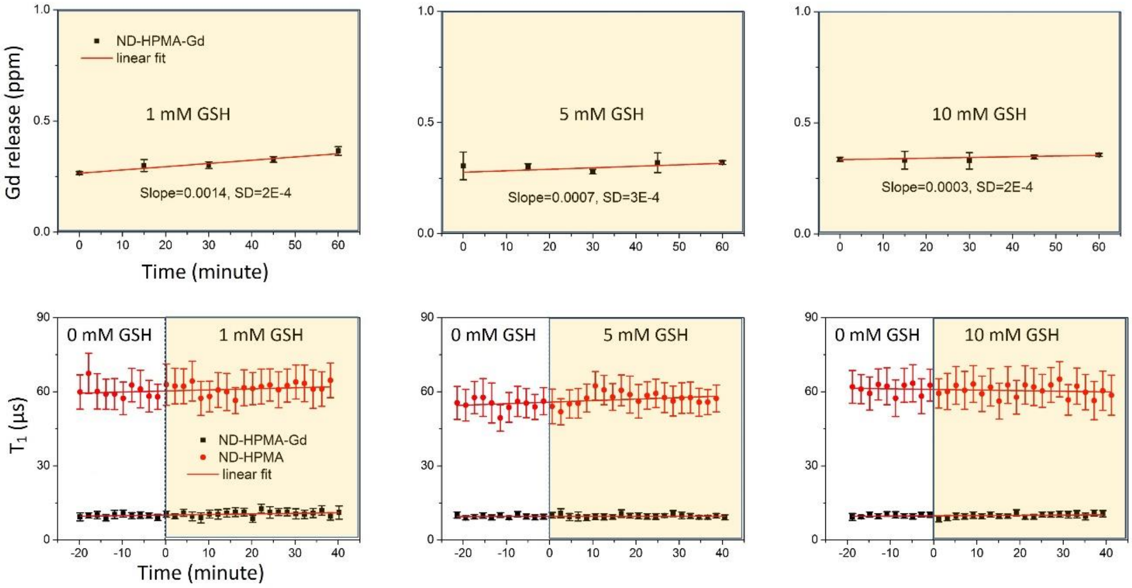

Supplementary Figure 5. The behavior of the control sample in buffers containing GSH. Release kinetics of Gd^{3+}-complex from ND-HPMA-Gd (non-cleavable) checked by ICP MS (upper panel) in different GSH buffers (1 mM, 5 mM and 10 mM GSH). Time-dependent ensemble measurement for T_1 (lower panel) of ND-HPMA and ND-HPMA-Gd (non-cleavable) in different GSH buffers (1 mM, 5 mM and 10 mM). The slope of all linear fit is approaching zero, indicating negligible change of Gd^{3+} complex within the measurement time period.

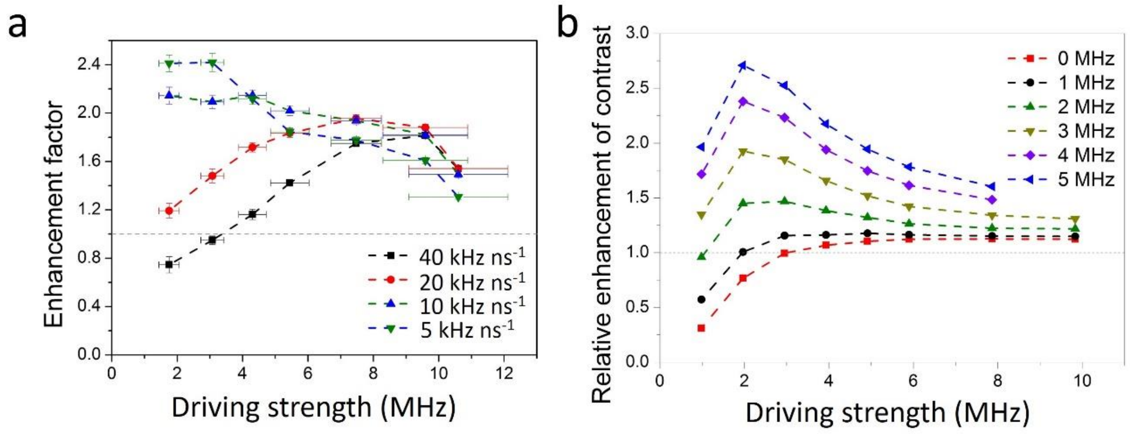

Supplementary Figure 6. Relative enhancement for chirp pulse versus a square pulse. (**a**) Comparison of experimentally obtained enhancement in sensitivity between T_1 relaxation measurement with linear chirp pulse over square pulse; the enhancement factor is calculated as the ratio of sensitivity obtained with linear chirp pulse to that with square pulse. The vertical error bars represent the standard deviations from 20 independent measurements with $\tau \ll T_1$, while the horizontal error bars represent the standard errors (95% confidence intervals) from Lorentzian fits (for Fourier transformed Rabi oscillation). (**b**) Simulation of relative enhancement of the probability to depopulate the NV $m_s = 0$ sublevel after applying linear chirp pulse over square pulse with different microwave driving strength.

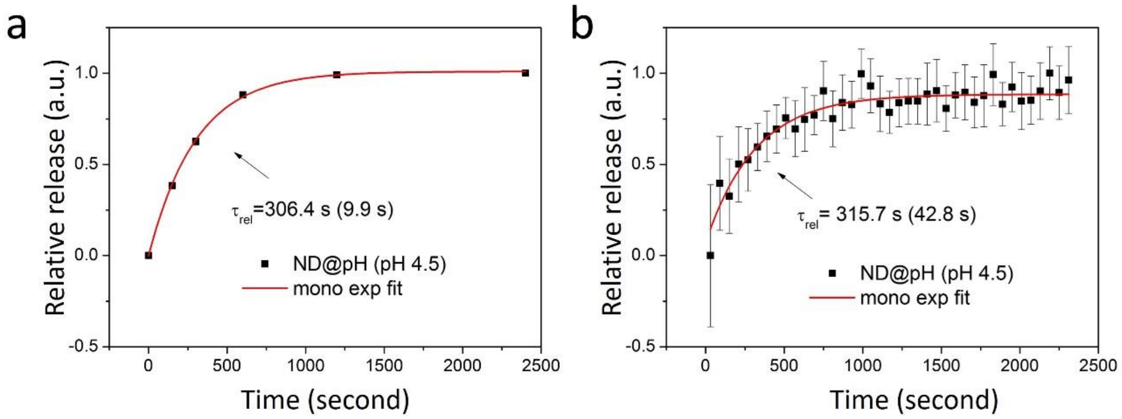

Supplementary Figure 7. Release kinetics of Gd^{3+} complexes. The results were obtained by (**a**) ICP MS and (**b**) ensemble T_1 measurement (ND@pH particles in pH 4.5 buffer). The standard deviations are stated in parentheses.

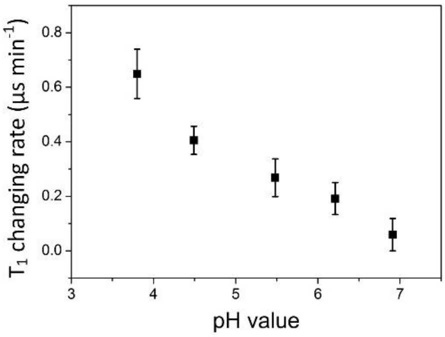

Supplementary Figure 8. Dependence of the fitted T_1 changing rate of ND@pH nanosensor on pH. Six T_1 measurement points (120 seconds per point) were linearly fitted to extract the actual T_1 change rate corresponding to a particular pH. Britton-Robinson buffers with same composition were used. Their conductivity was normalized using KCl to ensure environment with equal ionic strength for all measurements.

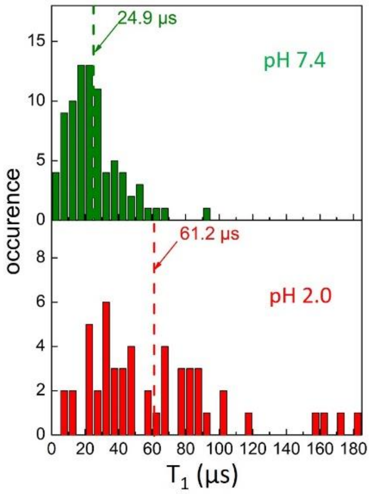

Supplementary Figure 9. Statistical view of ND@pH nanosensors in pH 7.4 and pH 2.0 buffers. After injecting the sample into a microfluidic chamber, the individual relaxation times for different adsorbed ND@pH nanosensors had been measured at pH 7.4 (green) and after changing to pH 2.0 (red).

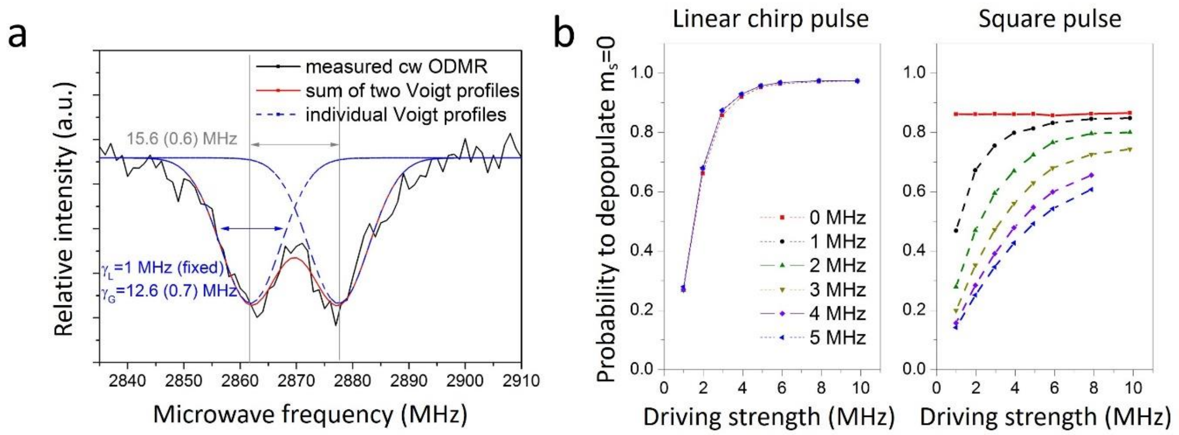

Supplementary Figure 10. Simulated NV spin flip probability induced by different pulse scheme. (**a**) A typical measured ODMR line for a randomly picked cluster of the ND-HPMA particles (black) excited with 45µW laser and weak microwave. The ODMR spectrum had been fitted by two Voigt profiles with a fixed Lorenzian linewidth of 1 MHz and a variable Gaussian linewidth (see below for used parameters). Both transitions in the measured ODMR line are quite broad and separated by around 16 MHz. (**b**) Simulated NV spin flip probability excited with a linear chirp pulse and square pulse plotted as a function of microwave driving strengths. The probability was calculated for an ensemble by averaging over all polar angle and assuming an inhomogeneous broadening of both ESR transitions with a variable value (color coded from 0 MHz (red) to 5MHz (blue)). Both transitions of ODMR line are set to split in average ~16 MHz to mimic the experimental situation in (**a**). The parameters of the chirp pulse had been set to typical values used under real experimental conditions (50 MHz sweep bandwidth with a 20 kHz ns^{-1} sweep speed).

Supplementary Methods

Chemical synthesis of Gd^{3+} complexes and diamond nanosensors

Chemicals and methods

3-Bromo-1-(trimethylsilyl)-1-propyne (**1**)[1], *t*Bu_3DO3A·HBr (**4**·HBr)[2], 1,6-bis(azido)-3,4-dithiahexane (**6**)[3], 10-({hydroxy[4-aminobenzyl]phosphoryl}methyl)-1,4,7,10-tetraazacyclododecane-1,4,7-triacetic acid (**7**)[4] and pent-4-ynehydrazide (**8**) (*via* methyl pent-4-ynoate[5]) were prepared according to published procedures. 6-Azido-hexan-2-one (**9**) was prepared analogously as published for related compounds[6]. Paraformaldehyde was filtered from aged aqueous formaldehyde solutions (Lachema) and dried in a desiccator over concentrated H_2SO_4. Other chemicals from commercial sources were used as received. Acetonitrile and dichloromethane were dried by distilling over P_4O_{10}.

NMR spectra were recorded on VNMRS300, Varian^{UNITY} INOVA 400 or Bruker Avance III 600 spectrometers. NMR chemical shifts are given in ppm, and coupling constants are reported in Hz. For ^{1}H and ^{13}C NMR measurements in D_2O, *t*BuOH was used as internal standard (δ_H = 1.25, δ_C = 30.29). For measurements in $CDCl_3$, TMS was used as internal standard (δ_H = 0.00, δ_C = 0.00). For ^{31}P NMR measurements, 85% aqueous H_3PO_4 was used as external reference (δ_P = 0.00). The abbreviations s (singlet), d (dublet), t (triplet), m (multiplet) and br (broad) are used to express signal multiplicities.

Mass spectra were measured on a Bruker Esquire 3000 mass spectrometer with electrospray ionization with ion-trap detection in both positive and negative modes. Mass signals are provided with their relative abundance to the strongest one. For Gd^{3+} complexes, only the one containing the most abundant isotope is stated. HR MS spectra were recorded on an LTQ Velos Pro or Orbitrap ELITE mass spectrometer by Thermo.

HPLC measurements were performed on a ReproSil Gold C8 5 μm 150×4.6 mm column with a flow rate of 1 ml·min^{-1} using UV-absorption detection at 210 nm and 256 nm. The following methods with linear v/v gradients of water/acetonitrile were used: method **A**: 5% ACN to 90% ACN with 10 ppm v/v trifluoroacetic acid (TFA) in 5 min, then 90% ACN with 10 ppm v/v TFA for 5 min; method **B**: 0% ACN to 10% ACN with 5 ppm v/v TFA in 10 min, then 10% ACN to 25% ACN with 5 ppm v/v

TFA in 10 min, then 25% ACN to 95% ACN with 5 ppm v/v TFA in 10 min, then 95% ACN to 99% ACN with 1 ppm v/v TFA in 5 min; method **C**: 0% ACN to 10% in 10 min, then 10% ACN to 25% ACN in 10 min, then 25% ACN to 95% in 10 min, then 95% ACN to 99% ACN in 5 min.

Thin-layer chromatography (TLC) was performed on TLC aluminium sheets silica gel 60 F254 (Merck). UV light or dipping in 2% $KMnO_4$/10% Na_2CO_3 or 5% aqueous $CuSO_4$ were used for detection.

Elemental analyses were performed at the Institute of Macromolecular Chemistry (Academy of Sciences of the Czech Republic, Prague).

Synthesis of redox sensitive complex $[Gd(L^1)]^-$–SS–N_3

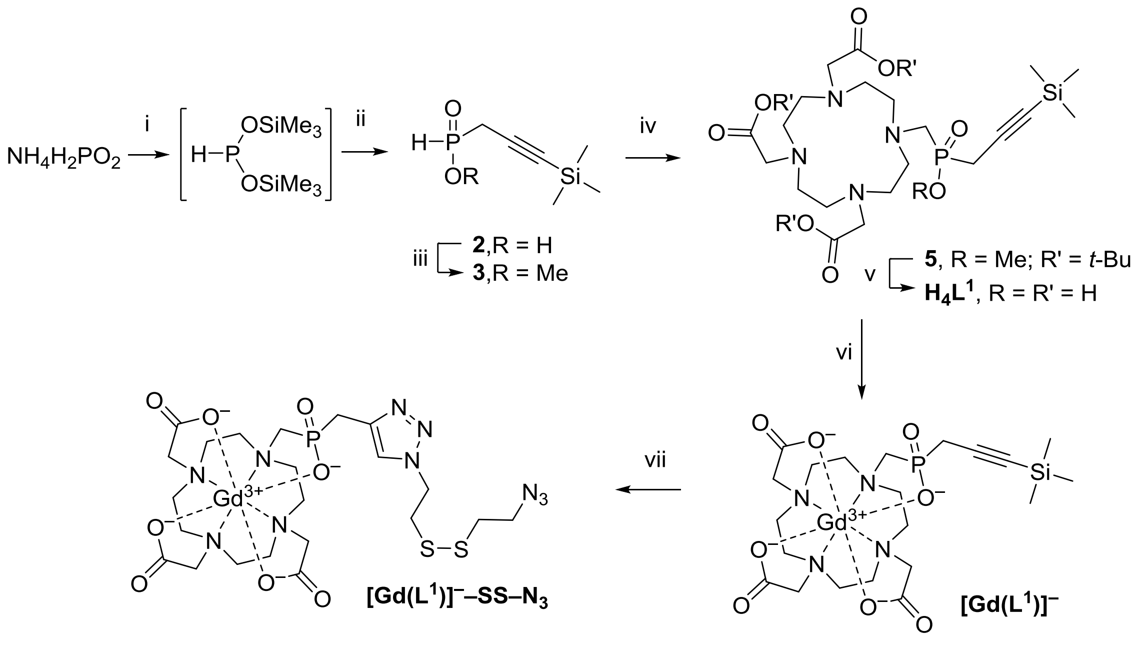

Reaction conditions and yields: i) Hexamethyldisilazane, under Ar, 105 °C, 12 h ii) 3-Bromo-1-(trimethylsilyl)-1-propyne (**1**)/CH_2Cl_2, under Ar, -10 °C to rt, 24 h, 54 % iii) methyl chloroformate, pyridine/CH_2Cl_2, reflux, 15 min, 97 % iv) *t*Bu_3DO3A·HBr (**4**·HBr), $(CH_2O)_n$/MeCN (dry), 65 °C, 3 days, ~90 % v) 1. 85% aq. HCOOH, 65 °C, 4 days, 2. 1,5% aq. HCl, rt, overnight, 78% vi) $GdCl_3·5H_2O$/aq. NH_4OH, pH 4,8, rt, overnight, ~60 % vii) 1,6-bis(azido)-3,4-dithiahexane (**6**), $CuSO_4$, NaF, Sodium Ascorbate/ THF:*i*PrOH:H_2O (1:1:2), rt, 12 h, ~50%

3-(Trimethylsilyl)prop-2-ynylphosphinic acid (2)

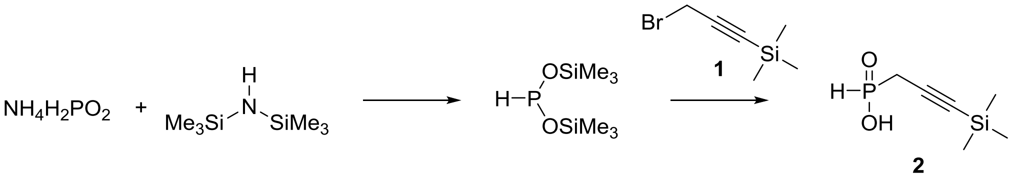

$NH_4H_2PO_2$ (5.0 g; 60 mmol) was securated in a 50 ml three-neck flask equipped with an argon inlet, a reflux condenser with vacuum trap and a septum. Hexamethyldisilazane (15 ml; 72 mmol) was added and the reaction mixture was stirred at 105 °C for 12 h under gentle flow of argon. In the course of the reaction, $NH_4H_2PO_2$ dissolved and ammonia evolved. The reaction mixture was then cooled to –10 °C and diluted with anhydrous dichloromethane (30 ml). Under an argon atmosphere, alkylation agent **1** (3.1 g; 16.2 mmol) was slowly added *via* syringe and the mixture was left to warm to RT under stirring. After 24 h, the reaction was terminated by addition of anhydrous EtOH (10 ml) and the reaction mixture was evaporated to dryness. The reaction mixture was co-evaporated two times with anhydrous EtOH (15 ml) and the remaining matter was dissolved in $CHCl_3$ (20 ml) and quickly washed with 3% HCl (2×20 ml). The combined aqueous phases were extracted with $CHCl_3$ (20 ml). The organic phases were combined, dried with Na_2SO_4 and volatiles were removed with rotary evaporator at 50 °C to yield (**2**) (1.54 g; 54 %) as a colorless oil.

The product contained ca 3% (by ^{31}P NMR) of bis(3-(trimethylsilyl)prop-2-ynyl)phosphinic acid, which does not react in the next step, and therefore, the crude **2** can be used without further purification. For analytic purposes, the compound **2** was purified by flash column chromatography on silica (EtOAc:MeOH = 5:4 v/v).

TLC: (EtOAc:MeOH = 5:4 v/v), R_f = 0.50, $KMnO_4$; ^{1}H NMR (299.94 MHz, $CDCl_3$): δ 0.17 (s, 9H, CH_3); 2.83 (dd, $^2J_{HP}$ = 20.1, $^3J_{HH}$ = 1.8, 2H, CH_2); 7.11 (d, $^1J_{HP}$ = 584, 1H, P–*H*); 10.9 (bs, O*H*); ^{13}C NMR (75.43 MHz, $CDCl_3$): δ –0,1 (s, *C*H_3); 24.6 (d, $^1J_{CP}$ = 84.5, *C*H_2); 86.8 (d, $^3J_{CP}$ = 7.5, C–*C*–Si); 99.4 (d, $^2J_{CP}$ = 8.7, P–C–*C*); ^{31}P NMR (121.42 MHz, $CDCl_3$): δ 27.8 (dt, $^1J_{HP}$ = 584, $^2J_{HP}$ = 20.0).

Methyl 3-(trimethylsilyl)prop-2-ynylphosphinate (3)

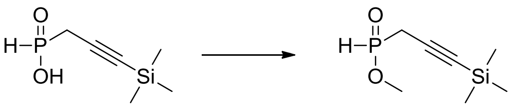

Phosphinic acid **2** (1.64 g; 9.31 mmol) was dried in a 100 ml flask by triple co-evaporation with anhydrous EtOH (5 ml). After evaporation, phosphinic acid was dissolved in anhydrous CH_2Cl_2 (19 ml), and methyl chloroformate (0.88 g; 9.31 mmol) was added. Anhydrous pyridine (0.74 g; 9.31 mmol) was added dropwise with stirring. After evolution of CO_2, the flask was equipped with a reflux condenser and the mixture was heated to reflux for 15 min. The reaction mixture was then cooled to RT and washed with 3% aqueous HCl (3×20 ml) and brine (20 ml). Combined aqueous phases were extracted with CH_2Cl_2 (2×20 ml) and organic phases were dried with Na_2SO_4, and solvent was removed with a rotary evaporator at 50 °C to yield methylester **3** (1.72 g; 97%) as a colorless liquid.

^{1}H NMR (299.94 MHz, $CDCl_3$): δ 0.12 (s, 9H, SiC*H*$_3$); 2.81 (d, $^2J_{HP}$ = 18.9, 2H, C*H*$_2$); 3.81 (d, $^3J_{HP}$ = 12.0, 3H, OC*H*$_3$); 7.16 (d, $^1J_{HP}$ = 585, 1H, P–*H*); ^{13}C NMR (75.43 MHz, $CDCl_3$): δ 0.3 (s, Si*C*H$_3$), 21.9 (d, $^1J_{CP}$ = 91.3, *C*H$_2$); 53.1 (d, $^2J_{CP}$ = 7.2, P–C–*C*); 89.9 (d, $^3J_{CP}$ = 8.5, C–*C*–Si); 93.4 (d, $^2J_{CP}$ = 10.9, O*C*H$_3$); ^{31}P NMR (121.42 MHz, $CDCl_3$): δ 31.5 (bd, $^1J_{HP}$ = 571.3).

Methyl ({4,7,10-tris[(*tert*-butyloxykarbonyl)methyl]-1,4,7,10-tetraazacyclododecane-1-yl}methyl)-3-(trimethylsilyl)prop-2-ynylphosphinate (5)

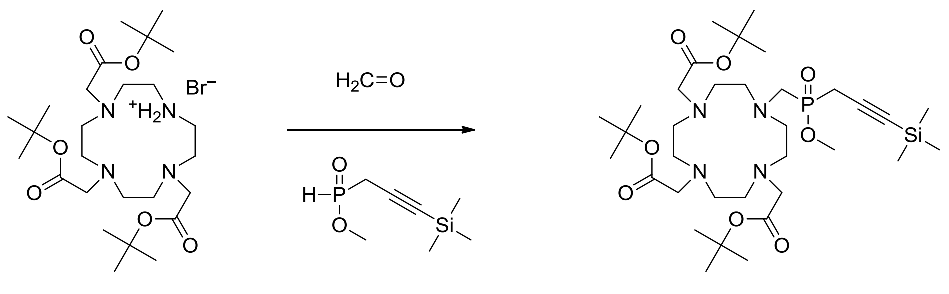

tBu_3DO3A·HBr (**4**·HBr, 1.000 g; 1.679 mmol) was placed into a 25 ml flask, and methyl ester **3** (336 mg; 1.763 mmol) and paraformaldehyde (53 mg; 1.763 mmol) were added under stirring. Anhydrous acetonitrile (10 ml) was then added, the flask was tightly closed, and the mixture was heated in an oil bath at 65 °C. The progress of the reaction was monitored with ^{31}P NMR spectra in 12-h intervals. A decrease in signal intensity of the starting compound **3** at 30 ppm (bd, $^{1}J_{HP}$ = 571 Hz) and increase in the product **5** signal at 43 ppm (s) was observed. In addition, approximately 3% of acid **2** was detected. Over time, the reaction mixture turned from a white suspension into pale brown clear solution. After 3 days, more than 95% of ester **3** had reacted, and solvent was removed with a rotary evaporator. The obtained oil was dissolved in minimal amount of hot MeOH, the solution was cooled, and the product was precipitated by addition of diethylether. The product **5** was collected by filtration (S3 frit), washed with diethylether (5 ml) and air-dried, affording 1.095 g (~90%) of off-white powder.

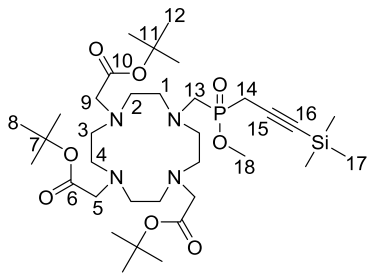

TLC: (EtOAc:EtOH:25% aq. NH_4OH = 60:33:7 v/v/v), R_f = 0.70, $KMnO_4$; ^{1}H NMR (299.94 MHz, $CDCl_3$): δ 0.07 (s, 9H, H-17); 1.40 (bs, 27H, H-8, H-12); 2.62 (d, $^{2}J_{HP}$ = 18.3, 2H, H-13); 2.60–3.60 (m, 24H, H-1-5, H-9, H-14); 3.75 (d, $^{3}J_{PH}$ = 10.8, 3H, H-18); ^{13}C NMR (75.43 MHz, $CDCl_3$): δ 0.1 (s, C-17); 23.2 (d, $^{1}J_{CP}$ = 84.8, C-14); 29.4 (s ,C-12); 30.6 (s, C-8); 51.2 (s, C-2); 51.8 (s, C-1,3); 52.2 (d, $^{2}J_{CP}$ = 97.8, C-13); 53.8 (s, C-4); 54.9 (s, C-9); 56.3 (s, C-5); 58.3 (d, $^{2}J_{CP}$ = 7.2, C-18); 84.6 (s, C-7); 86.1 (s, C-11); 92.1 (d, $^{3}J_{CP}$ = 8.9, C-16); 98.3 (d, $^{2}J_{CP}$ = 12.1, C-15); 170.2 (s, C-6); 174.2 (s, C-10); ^{31}P NMR (121.42 MHz, CD_3OD): δ 44.3 (bs); MS(+): 717.7 ($[M+H]^+$).

10-({Hydroxy[3-(trimethylsilyl)prop-2-ynyl]phosphoryl}methyl)-1,4,7,10-tetraazacyclododecane-1,4,7-triacetic acid (H_4L^1)

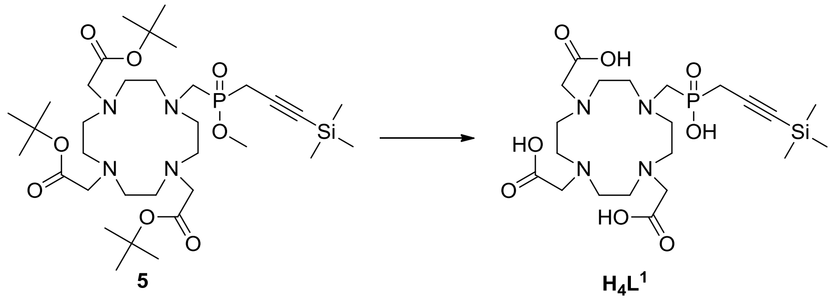

Compound **5** (1.000 g; 1.395 mmol) was dissolved in 85% formic acid (10 ml) in a 25-ml flask equipped with a magnetic stirring bar. The flask was closed, and the reaction mixture was heated in an oil bath at 65 °C for 4 days. Solvent was removed *in vacuo*, and the resulting oil was co-evaporated with water (3×5 ml) to remove the remaining formic acid. The obtained pale brown oil was dissolved in 1.5% aqueous HCl (10 ml) and stirred at RT overnight. Solvent was removed with a rotary evaporator. The resulting oil was dissolved in minimal amount of hot MeOH, the solution was cooled, and the product was precipitated by addition of diethylether. The product was collected by filtration on S3 frit, washed with diethylether (5 ml), dried in a vacuum and left to equilibrate at ambient conditions for three weeks. Product $\mathbf{H_4L^1}$ (0.694 g; 78%) was obtained as dihydrochloride sesquihydrate in form of an off-white powder.

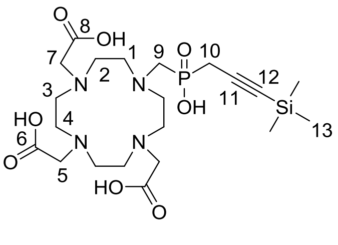

TLC: (EtOH:10% aq. AcOH = 8:2 v/v), R_f = 0.40; (EtOAc:EtOH:25% aq. NH_4OH = 60:33:7 v/v), R_f = 0.10, $CuSO_4$ or $KMnO_4$; ^{1}H NMR (299.94 MHz, D_2O): δ 0.07 (s, 9H, H-13), 2.64 (t, $^2J_{PH}$ = 18.6; 2H, H-10) 2.75–3.95 (bm, 24H; H-1-5, H-7, H-9); ^{13}C NMR (75.43 MHz, D_2O): δ 1.5 (s, C-13); 26.5 (d, $^1J_{CP}$ = 91.7; C-10); 50.5 (s, C-2); 51.6 (s, C-1,3); 51.8 (d, $^2J_{CP}$ = 98.3; C-9); 53.4 (s, C-4); 54.3 (s, C-7); 55.7 (s, C-5); 91.3 (d, $^3J_{CP}$ = 7.2, C-12); 102.8 (d, $^2J_{CP}$ = 10.5; C-11); 172.2 (s, C-6); 177.1 (s, C-8); ^{31}P

NMR (121.42 MHz, D_2O): δ 21.0–26.5 (br s); EA: calc. for $\mathbf{H_4L^1}$·2HCl·1.5H_2O, $C_{21}H_{44}Cl_2N_4O_{9.5}PSi$, *M* = 634.56: C 39.76; H 6.99; N 8.83; Cl 11.18; found: C 40.03; H 7.02; N 8.64; Cl 11.23; MS(+): 535.1 (51$[M+H]^+$); 557.1 (100$[M+Na]^+$); 573.1 (50$[M+K]^+$); MS(–): 532.9 (100$[M–H]^-$); 570.9 (21$[M+K–H]^+$).

$(NH_4)[Gd(L^1)]$ complex

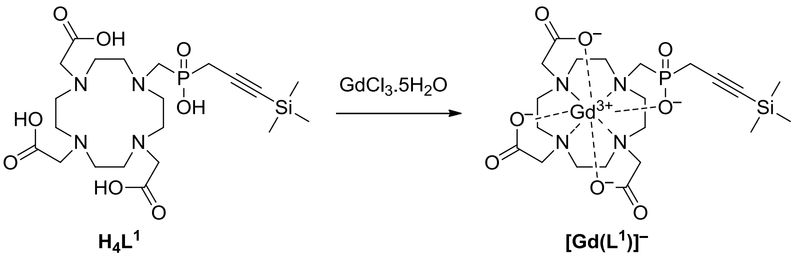

$GdCl_3$·5H_2O (158 mg, 0.60 mmol) was added to a solution of $\mathbf{H_4L^1}$ (350 mg, 0.55 mmol) in water (10 ml) with stirring. The pH of the solution was adjusted to 4.8 with 5% aqueous NH_3 and the mixture was left at RT overnight. Solvent was then evaporated *in vacuo*, and the product was purified by reversed-phase chromatography (40 g YMC DispoPackAT 25-ODS cartridge) with a water–acetonitrile gradient and 10 ppm trifluoroacetic acid modifier. Fractions containing the product were collected and lyophilized to obtain $(NH_4)[Gd(\mathbf{L^1})]$ (253 mg, ~60% yield) as a white powder.

MS(+): 712.1 (66$[[Gd(\mathbf{L^1})]+H+Na]^+$); 734.1 (100$[[Gd(\mathbf{L^1})]+2Na]^+$); 750.0 (97$[[Gd(\mathbf{L^1})]+Na+K]^+$); MS(–): 615.9 (15$[[Gd(\mathbf{L^1})]–TMS]^-$); 688.0 (100$[Gd(\mathbf{L^1})]^-$); HPLC: Program **A**: Retention time = 5.97 min.

Na[Gd(L^1)]–SS–N_3 complex

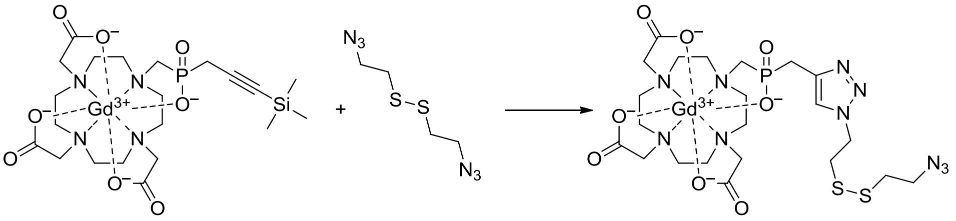

[Gd(L^1)]⁻ **6** **[Gd(L^1)]⁻–SS–N_3**

(NH_4)[Gd(**L**^{1})] (250 mg, 0.36 mmol) was dissolved in a mixture of water (10 ml), *i*PrOH (5 ml) and THF (5 ml) in a 50-ml flask. Then 1,6-bis(azido)-3,4-dithiahexane (456 mg, 2.23 mmol), 1 M aqueous NaF (450 μl) and 1 M aqueous $CuSO_4$ (90 μl) were added, and the flask was sealed with a septum. Using a needle, a gentle stream of argon was passed through the reaction mixture for 3 min with sonication. Then, 1 M aqueous sodium ascorbate (225 μl) was added, and the reaction mixture was stirred for 12 h at RT under an argon atmosphere. The solvent was removed with a rotary evaporator, and the product was purified by reversed-phase chromatography (40 g YMC DispoPackAT 25-ODS cartridge) with a water–acetonitrile gradient and 10 ppm trifluoroacetic acid modifier. Fractions containing the product were collected and lyophilized to obtain Na[Gd(**L**^{1})]–SS–N_3 (153 mg, approx. 50% yield) as a yellowish sticky solid.

MS(+): 822.2 ([[Gd(**L**^{1})]–SS–N_3+2H]$^+$); MS(–): 819.1 ([[Gd(**L**^{1})]–SS–N_3]$^-$); HRMS (ESI): calcd. for: $C_{33}H_{37}O_8N_{10}GdPS_2$ ([[Gd(**L**^{1})]–SS–N_3+2H]$^+$): 822.12104; found: 822.12158; calcd. for: $C_{33}H_{36}O_{10}N_{10}GdNaPS_2$ ([[Gd(**L**^{1})]–SS–N_3+H+Na]$^+$): 844.10298; found: 844.10355; HPLC: Program **B**: Retention time = 18.6 min.

Synthesis of pH sensitive complex $[Gd(L^2)]^{-}$–NHN–N_3

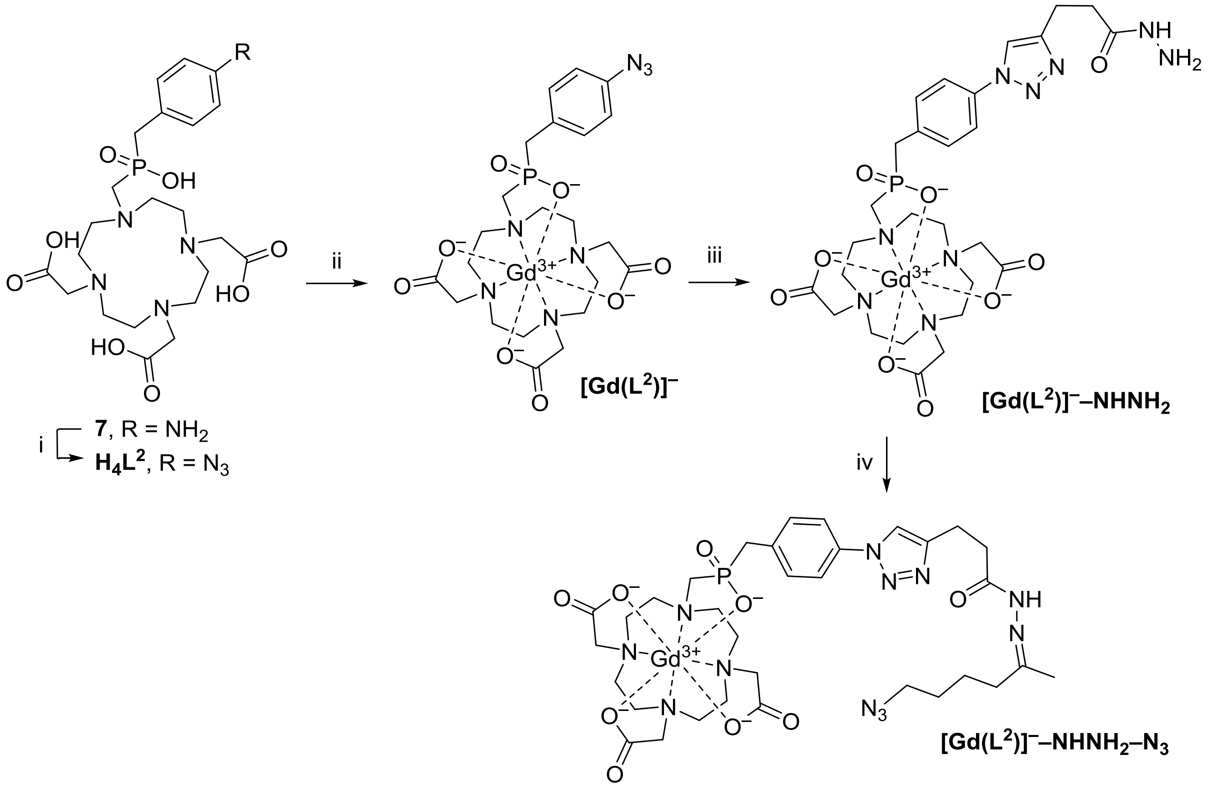

Reaction conditions and yields: i) 1. $NaNO_2$/aq. HCl 0 °C, 25 min; 2. NaN_3/aq. HCl 0 °C, 25 min, 70 % ii) $GdCl_3 \cdot 5H_2O$/aq. NH_4OH, pH 9, rt, overnight, ~70 % iii) pent-4-ynehydrazide (**8**), $CuSO_4$, Sodium Ascorbate/ THF:H_2O (1:1), rt, overnight, ~60 % iv) 6-azido-hexan-2-one (9), AcOH, $MgSO_4$/MeOH (dry), reflux, 3 days, ~30 %

10-({Hydroxy[4-azidobenzyl]phosphoryl}methyl)-1,4,7,10-tetraazacyclododecane-1,4,7-triacetic acid (H_4L^2)

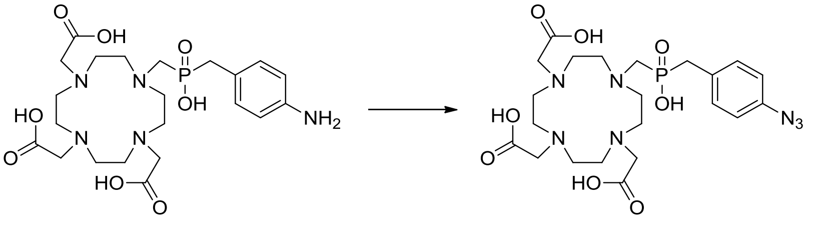

7 **H_4L^2**

A solution of 10-({hydroxy[4-aminobenzyl]phosphoryl}methyl)-1,4,7,10-tetraazacyclododecane-1,4,7-triacetic acid tetrahydrochloride (**7**·4HCl, 653 mg; 0.97 mmol) in a mixture of deionized water (5 ml) and 6 M HCl (1 ml) in a 25-ml flask was cooled to 0 °C. Then, $NaNO_2$ (95 mg; 1.38 mmol) was slowly added, and the reaction mixture was stirred at 0 °C for 25 min. NaN_3 (104 mg; 1.60 mmol) was then slowly added, and the reaction mixture was stirred at 0 °C for another 25 min. The reaction

mixture was placed in a rotary evaporator and twice co-evaporated with water (3 ml) to remove excess HCl. The product was purified by reversed-phase chromatography (40 g YMC DispoPackAT 25-ODS cartridge) with a water–acetonitrile gradient and 10 ppm trifluoroacetic acid modifier. Fractions containing the product were collected, lyophilized and left for three weeks to equilibrate in ambient air in the dark to obtain (**H_4L^2**) (495 mg; 70% yield) as white powder, which turns brown when exposed to light.

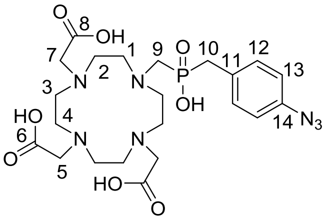

TLC: (EtOH:25% aq. NH_4OH = 8:2 v/v), R_f = 0.25, UV, $KMnO_4$ or $CuSO_4$; ^{1}H NMR (299.94 MHz, DMSO-d_6): δ 2.97–3.60 (bm, 24H; H-1-4, H-9, H-10); 3.64 (bm, 2H, H-5); 4.08 (bm, 4H, H-7); 7.03 (d, $^3J_{HH}$ = 7.9; 2H, H-13); 7.29 (d, J = 7.9 Hz, 2H, H-13); ^{13}C NMR (75.43 MHz, DMSO-d_6): δ 36.5 (d, $^1J_{CP}$ = 85.6; C-10); 49.9 (s, C-2); 50.8 (s, C-4); 51.3 (s, C-1); 50.6 (d, $^2J_{CP}$ = 105.1; C-9); 51.8 (s, C-3); 54.1 (s, C-5); 54.8 (s, C-7); 119.8 (d, $^4J_{CP}$ = 1.6; C-13); 130.2 (d, $^2J_{CP}$ = 10.0; C-11); 132.5 (d, $^3J_{CP}$ = 4.8; C-12); 138.3 (s, C-14); 170.1 (s, C-8); 172.6 (s, C-6); ^{31}P NMR (121.42 MHz, DMSO-d_6): δ 38.1 (s); EA: calc. for **H_4L^2**·3.4HCl·3H_2O, $C_{22}H_{43.4}Cl_{3.4}N_7O_{11}P$, M = 733.53: C 36.02; H 5.96; N 13.37, Cl 16.43; found: C 36.06; H 5.74; N 12.87, Cl 16.49; MS(+): 555.9 (100[M+H]$^+$); 577.9 (20[M+Na]$^+$); 593.9 (16[M+K]$^+$); MS(–): 553.7 ([M–H]$^-$); HPLC: Program **A**: Retention time = 5.79 min.

$(NH_4)[Gd(L^2)]$ complex

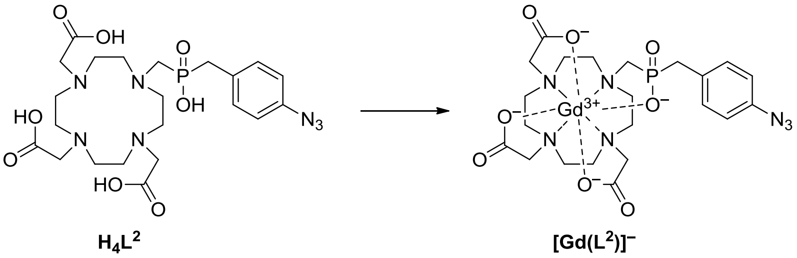

H_4L^2 **$[Gd(L^2)]^-$**

$GdCl_3 \cdot 5H_2O$ (126 mg, 0.49 mmol) was added to a solution of $\mathbf{H_4L^2}$ (300 mg, 0.41 mmol) in water (6 ml) with stirring. The pH of the solution was adjusted to 9.0 with 3% aqueous NH_4OH, and the mixture was left at RT overnight. The solvent was removed with a rotary evaporator, and the resulting solid was purified by flash chromatography (Buchi Sepacore® Silica 25 g cartridge) with an EtOH:25% aqueous NH_4OH (10:1 v/v) mobile phase. Fractions containing the product were collected and dried on a rotary evaporator. The product $(NH_4)[Gd(\mathbf{L}^2)]$ (227 mg, ~70% yield) was obtained after lyophilization from water (10 ml) as a pale powder that turns dark when exposed to light.

TLC: (EtOH:25% aq. NH_4OH = 5:1 v/v), $R_f = 0.65$, UV, $KMnO_4$; MS(–):707.4 ($(Gd(\mathbf{L}^2))^-$); HPLC: Program **A**: Retention time = 5.69 min.

$(NH_4)[Gd(L^2)]^-$–$NHNH_2$ complex

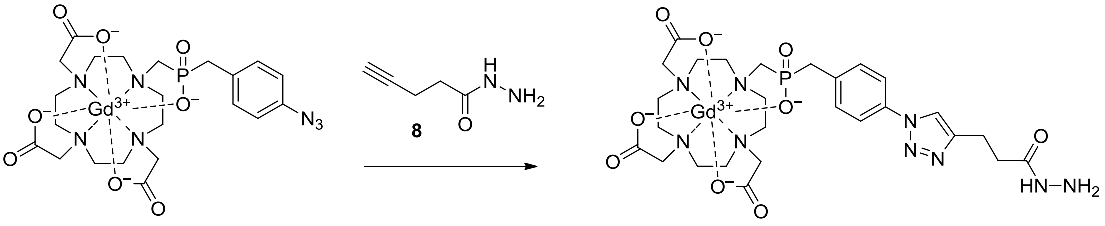

$[Gd(L^2)]^-$ **$[Gd(L^2)]^-$–$NHNH_2$**

$(NH_4)[Gd(\mathbf{L}^2)]$ (203 mg; 0.28 mmol) and pent-4-ynehydrazide **8** (62 mg, 0.55 mmol) were dissolved in a mixture of water (1.5 ml) and THF (1.5 ml) in a 4-ml vial equipped with a septum and magnetic stirring bar. Then, 1 M aqueous $CuSO_4$ (32 μl) was added, and the mixture was bubbled under a brisk stream of argon for 3 min. Subsequently 1 M aqueous sodium ascorbate (100 μl) was added, and the solution was stirred at RT overnight in the dark. The solvent was removed with a rotary evaporator, and the resulting solid was purified with reversed-phase chromatography (40 g YMC DispoPackAT 25-ODS cartridge) with a water–acetonitrile gradient and 10 ppm trifluoroacetic acid modifier. Fractions containing the product were collected and lyophilized to obtain $(NH_4)[Gd(\mathbf{L}^2)]$–$NHNH_2$ (154 mg, ~60% yield).

MS(+): 821.5 (33[[Gd(**L**^{2})]–$NHNH_2$+2H]$^+$); 844.6 (66[[Gd(**L**^{2})]–$NHNH_2$+H+Na]$^+$); 866.6 (100[[Gd(**L**^{2})]–$NHNH_2$+H+K]$^+$); MS(–): 820.5 ([[Gd(**L**^{2})]–$NHNH_2$–H]$^-$); HPLC: Program **B**: Retention time = 10.5 min.

(NH_4)[Gd(L^2)]–NHN–N_3 complex

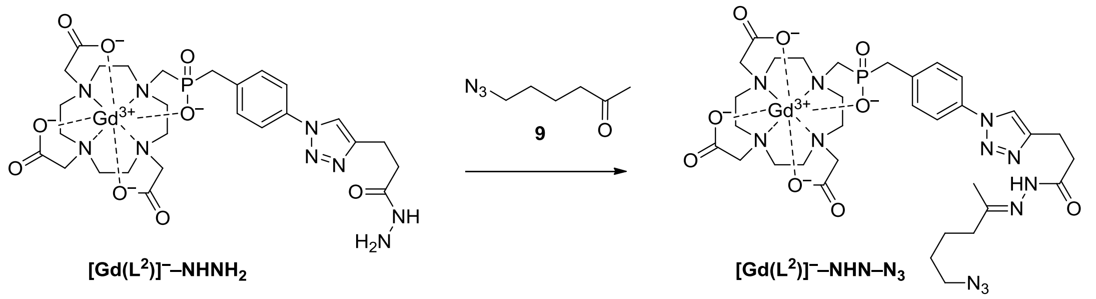

(NH_4)[Gd(**L**^{2})]–$NHNH_2$ (100 mg; 0.12 mmol) and 6-azido-hexan-2-one **9** (137 mg; 0.97 mmol) were dissolved in anhydrous MeOH (8 ml) in a 25-ml flask equipped with a reflux condenser with a chlorcalcium tube. Glacial acetic acid (22 mg; 0.37 mmol) and anhydrous $MgSO_4$ (1.00 g; 8.33 mmol) were added, and the reaction mixture was heated to reflux under chlorcalcium tube for 3 days. The solvent was removed with a rotary evaporator, and the resulting solid was purified with reversed-phase chromatography (40 g YMC DispoPackAT 25-ODS cartridge) with a water–acetonitrile gradient. Fractions containing the product were combined and lyophilized to obtain **(NH_4)[Gd(L^2)]–NHN–N_3** (33 mg, ~30% yield).

MS(–): 944.3 ([M–H]$^-$); HRMS (ESI): calcd. for: $C_{33}H_{47}O_9N_{12}GdP$ ([[Gd(**L**^{2})]–NHN–N_3]$^-$): 944.25731; found: 944.25711; HPLC: Program **C**: Retention time = 18.9 min.

Preparation of ND-HPMA-Gd conjugates

Nanodiamonds (NDs) (MSY 0–0.05, Microdiamant, Switzerland) were oxidized by air in a furnace at 510 °C for 4 h; treated with a mixture of HF and HNO_3 (2:1) at 160 °C for 2 days; and washed with water, 1 M NaOH, 1 M HCl and water. Purified ND powder was irradiated in an external target holder for 21 hours with a 16.6 MeV electron beam (1.25 × 10^{19} particles cm^{-2}) from an MT-25 microtron.

The irradiated material was annealed at 900 °C for 1 h and subsequently oxidized for 4 h at 510 °C. The resulting powder was again treated with a mixture of acids and washed with NaOH, HCl and water, providing a colloidal solution of ND-COOH.

Poly(vinylpyrrolidone) (MW = 10,000, 136 mg) was dissolved in water (120 ml) and sonicated for 10 min in an ultrasonic bath. ND-COOH colloid (24 ml, 2 mg ml^{-1}, filtered using a 0.4 µm GMF filter) was added, and the mixture was stirred for 24 h. The colloid was then concentrated by centrifugation. NDs (in approximately 3 ml solvent) were resuspended in ethanol (48 ml). Tetraethyl orthosilicate (360 µl) and 3-(trimethoxysilyl)propylmethacrylate (120 µl) were added, and the mixture was sonicated for 20 s in an ultrasonic bath. Ammonia (25%, 2 ml) then was added. The reaction mixture was stirred for 14 h. The product was purified by centrifugation, washed with methanol and transferred to 0.3 ml of DMSO using a rotary evaporator. (2-Hydroxypropyl)methacrylamide (HPMA) (735 mg), N-propargyl acrylamide (315 mg) and 2,2-azobis(2-methylpropionitrile) (AIBN, 300 mg) were dissolved in DMSO (3 ml). The mixture was filtered using a 0.4 µm glass microfiber microfilter. Methacrylate-terminated NDs (48 mg) dispersed in 0.3 ml DMSO were added. The reaction proceeded for 3 days under argon at 55 °C. The particles were purified by centrifugation with methanol. Alkyne-modified HPMA-coated NDs were decorated with azide-modified Gd^{3+} complexes using Cu(I)-catalyzed azide-alkyne cycloaddition (CuACC). HPMA-coated NDs (10 mg in a final reaction volume of 12.8 ml 50 mM HEPES buffer, pH 7.4) were mixed with non-cleavable Gd^{3+} complex (**$NH_4[Gd(L^2)]$**), cleavable Gd complex with hydrazone (**$NH_4[Gd(L^2)]$–NHN–N_3**) or a disulfide linker (**$Na[Gd(L^1)]$–SS–N_3**) in final concentrations of 0.96, 1.92 and 2.4 mM, respectively, pre-mixed 0.32 mM $CuSO_4$ and 0.64 mM tris(3-hydroxypropyltriazolylmethyl)amine (THPTA) ligand and a freshly prepared solution of sodium ascorbate (5 mM). The reaction mixture was well-sealed, left for 1 h with no stirring and washed by centrifugation with water (Gd^{3+} conjugates with non-cleavable, ND-HPMA-Gd, and disulfide, ND@redox, linkers) or methanol (Gd^{3+} conjugates with hydrazone linker, ND@pH). The resulting nanosensors were stored in water (ND-HPMA-Gd and ND@redox) or in dry methanol (ND@pH) at 4 °C.

Characterization of ND-polymer-Gd nanosensors

The stability and surface charge of HPMA-coated NDs with Gd^{3+} complexes were tested in solutions later used for further experiments: 50 mM citric acid (pH 2.0), 50 mM acetate buffer (pH 4.5), 50 mM HEPES buffer (pH 7.4), 50 mM TRIS buffer (pH 8.5) and 10× concentrated PBS. Dynamic light scattering (DLS) and zeta potential were recorded with a Zetasizer Nano ZS system (Malvern Instruments) at 37 °C. Sample concentrations were 0.1 mg ml^{-1}.

To prepare the samples for transmission electron microscopy (TEM), carbon-coated copper grids were placed into a UV-ozonizing chamber (UV/Ozone Pro Cleaner Plus, Bioforce Nanosciences) for 15 min. Then, a droplet of jetPEI (Polyplus tranfection, cat no. 101-10) was placed on the grid. After 10 min incubation, it was removed with a piece of tissue. Then, a droplet of aqueous solution of NDs (0.05 mg ml^{-1}) was placed on the grid, and after 3 min incubation, liquid was removed with a piece of tissue. TEM pictures were captured using a JEOL JEM-1011 electron microscope operated at 80 kV.

Total amounts of Gd^{3+} complexes conjugated to HPMA-coated NDs were estimated using inductively coupled plasma atomic emission spectroscopy (ICP AES). The analysis was performed using a Spectro Arcos SOP ICP AES spectrometer, power 1450 W, sample flow 2 ml min^{-1}.

Kinetics of release of Gd^{3+} complex from the conjugate

ND@pH: A 5-µl aliquot of NDs colloid (20 mg ml^{-1} in water) was diluted with 95 µl buffer (either 50 mM citrate, pH 2.0; 50 mM acetate, pH 4.5 or 50 mM HEPES, pH 7.4). The mixture was incubated for a certain time (30 s, 1, 2, 4 and 60 min for pH 2.0; 2.5, 5, 10, 20, 40 min and 8 h for pH 4.5; and 2, 6, 12 and 24 hours for pH 7.4) at 37 °C. The cleavage reaction was then stopped by addition of HEPES buffer (300 mM, pH 8.0) to the mixture (100 µl for pH 2.0, 20 µl for pH 4.5). NDs were centrifuged (10 min 55,000 rcf, 4 °C), and the supernatant was removed and diluted with 2% HNO_3 for ICP MS measurements. A control representing time 0 s was set up with the opposite order of mixing: first, the NDs colloid was diluted in HEPES buffer, then cleavage buffer (acidic conditions) was added. The non-cleavable ND-HPMA-Gd conjugate was used as a control at the 0, 15, 30, 45 and

60 minutes and processed under the same conditions. All samples were prepared in duplicate. Concentrations of Gd^{3+} complexes in solutions were analyzed in duplicate (performed with two independent samples, where each was measured twice (4 results)) using an ICP MS 7700 (Agilent Technologies) instrument.

ND@redox: A 5-µl aliquot of NDs colloid (20 mg ml^{-1} in water) was diluted with 95 µl buffer (50 mM HEPES buffer, pH 7.4, or 50 mM TRIS, pH 8.5). A solution of glutathione (final concentrations of 1, 5 and 10 mM) was added. The mixture was incubated for a certain time (2, 5, 10, 30 and 60 min) at 37 °C under inert conditions. The cleavage reaction was then stopped by addition of 6-maleimidohexanoic acid (diluted in 10% DMSO in 0.5 M phosphate buffer, pH 7.0, final concentration 100 mM). NDs were centrifuged (10 min 55,000 rcf, 4 °C), and the supernatant was removed and diluted with 2% HNO_3 for ICP MS measurements. A control representing time 0 s was set up with the opposite order of mixing: 6-maleimidohexanoic acid was first added to NDs colloid diluted in buffer and then glutathione was added. The non-cleavable ND-HPMA-Gd conjugate was used as a control at the 0, 15, 30, 45 and 60 minutes and processed under the same conditions. All samples were prepared in duplicate. Concentrations of Gd^{3+} complexes in solutions were analyzed in duplicate (performed with two independent samples, where each was measured twice (4 results)) using an ICP MS 7700 (Agilent Technologies) instrument.

Preparation of Britton-Robinson buffers (measurements in Supplementary Figure 8)

Britton-Robinson buffers (with equal composition and ionic strength) were prepared according to the established procedure: 0.5 M Britton-Robinson buffer at pH 3.29 was prepared (by mixing 0.0667 g of NaOH, 0.0664 ml CH_3COOH, 0.114 ml H_3PO_4, 0.103 g H_3BO_3 and 1.729 g KCl). For T_1 measurements, stock buffer was diluted to 50 mM concentration and pH was adjusted to required pH (3.8, 4.5, 5.5, 6.2 and 6.9) by NaOH. The conductivity of all solutions was adjusted to the same value (6.40 mS) by addition of KCl.

Theoretical model of NV relaxometry influenced by the release of Gd^{3+} complex

The total NV relaxation rate is:

$$\Gamma_{total} = \Gamma_{int} + \Gamma_{external} \quad (1)$$

The intrinsic decay rate Γ_{int} is attributed to spin noise (impurities) in the diamond lattice like Orbach or Raman process[7]. The external part $\Gamma_{external}$ is attributed to the randomly distributed Gd^{3+} complex locating in the polymer shell surrounding ND, and can be written as[8]:

$$\Gamma_{extrernal} = 3g_{NV}\mu_B \sum_i \frac{g_i\mu_i}{\hbar^2} < B_i^2 > \frac{\tau_{c_i}}{1+\omega_0^2\tau_{c_i}{}^2}$$

(2)

Where g_i is the g-factor, μ_i the magneton and τ_{c_i} is the typical correlation time of the spin species with index i.

As in equation (1) the decay rates just sum up, we can also combine several decay channels that are kept constant over time. We therefore consider the sample ND-HPMA (without Gd^{3+} complex) has an intrinsic relaxation time, and add another decay channel induced by Gd^{3+} for other samples (with Gd^{3+} complex).

Starting with a spin S_j placed at the distance r_j to the NV to derive an expression for $< B_i^2 >$, the spin creates a time fluctuating magnetic field B_j at the position of the NV:

$$\mathbf{B}_j = \frac{\mu_0}{4\pi} g_j \mu_j \cdot \frac{1}{r_j^3}\left(\mathbf{S}_j - \frac{3(\mathbf{S}_j \cdot \mathbf{r}_j)\cdot \mathbf{r}_j}{r_j^2}\right) \quad (3)$$

Weak magnetic field components along the NV quantization axis (z) only lead to a detuning off the NV resonance, but don't introduce a change in the spin level populations, as the x and y components will do. Therefore we can neglect the z-component of the B field:

$$B_{\perp,j}{}^2 = < B_{x,j}^2 > + < B_{y,j}^2 > \quad (4)$$

We assume the spin to be in a purely mixed state. Its density matrix ρ can be written as:

$$\rho = \frac{1}{2S+1} \mathrm{E}_{2S+1} \quad (5)$$

Than $B_{\perp,j}{}^2$ can be expressed as:

$$B_{\perp,j}^{\ 2} = Tr\left(\rho B_{x,j}^2\right) + Tr\left(\rho B_{y,j}^2\right) = \left(\frac{\mu_0}{4\pi} g_e \mu_B\right)^2 \cdot C_s \cdot \frac{2+3\sin^2(\alpha_j)}{r_j^6} \quad (6a)$$

with:

$$C_s = \frac{1}{2S+1}\sum_{m=-S}^{S} m^2 = \frac{S(S+1)}{3} \quad (6b)$$

In the following we consider spins randomly distributed in a layer with thickness δd and the volume density of σ_i on the surface of a spherical diamond with diameter d_o. The total magnetic field experienced by the NV center for all spin species can then be summarized in spherical coordinates:

$$< B_i^2 >= \sum_j B_{\perp,j}^2 = \left(\frac{\mu_0}{4\pi} g_i \mu_i\right)^2 \cdot C_s \cdot \sigma_i \int_{\frac{d_o}{2}}^{\frac{d_0}{2}+\delta d} dr' \int_0^{2\pi} d\phi \int_0^{\pi} d\theta \sin\theta \, \frac{2+3\sin^2(\alpha(\theta,\phi))}{r(r\prime,\delta r,\theta,\phi)^4} \quad (7a)$$

With:

$$\sin^2\alpha = \sin^2\theta \quad (7b)$$

And

$$r = \sqrt{r'^2 - \delta r^2 \sin^2\theta} + \delta r \cos\theta \quad (7c)$$

When moving δr from the center of a spherical shaped nanodiamond along the NV spin quantization axis (parallel to the NV axis).

If the NV is moved on a path perpendicular to its quantization axis, then $\sin^2\alpha$ and r have the following expression[8]:

$$\sin^2\alpha = \cos^2\theta + \sin^2\theta \sin^2\phi \quad (7d)$$

And

$$r = \sqrt{r'^2 - \delta r^2 \sin^2\theta} + \delta r \cos\theta \quad (7e)$$

The total fluctuation rate of the Gd^{3+} complex is $R = 1/\tau_c = R_{dip} + R_{vib}$where R_{dip} is due to the inter bath dipolar coupling while R_{vib} is caused by intrinsic vibrational spin relaxation of the Gd^{3+}

complex in solution. In current study, we adapted the typical value of $R_{vib} = 50\,GHz$ for Gd^{3+} complex[9].

In the following, we calculate the R_{dip}:

The spin-spin interaction of two spins k and l can be describe by the following Hamiltonian:

$$\mathbf{H}_{kl} = \frac{\mu_0}{4\pi} \mathrm{g_k}\mu_\mathrm{k} \cdot \mathrm{g_l}\mu_\mathrm{l} \frac{1}{\mathrm{r}_{kl}^3}\left(\mathbf{S_k S_l} - \frac{3(\mathbf{S_k}\cdot\mathbf{r_{kl}})\cdot(\mathbf{S_l}\cdot\mathbf{r_{kl}})}{\mathrm{r}_{kl}^2}\right) \quad (8)$$

And is connected to the interaction rate $R_{dip} = 1/\tau_c^{dip}$ with:

$$\hbar R_{dip} = \sqrt{\sum_{k\neq l} < H_{kl}^2 >} \quad (9)$$

By using (8) one finds[8]:

$$< H_{kl}^2 >= \left(\frac{\mu_0}{4\pi} \mathrm{g_k}\mu_\mathrm{k} \cdot \mathrm{g_l}\mu_\mathrm{l}\right)^2 \cdot 6C_s^2 \cdot \frac{1}{r_{kl}^6} \quad (10)$$

Spins in a thick layer:

For a layer with height of δd consisting of spins with the density σ we use the following approximation:

$$\sum_{k\neq l} \frac{1}{r_{kl}^6} \approx \sigma \int_0^{2\pi} d\phi \int_{d_{min}}^{\delta d - d_{min}} dz \int_{r_{min}}^{\infty} dr\ \frac{r}{(r^2+z^2)^3}\ = 2\pi\sigma \int_{d_{min}}^{\delta d - d_{min}} dz \int_{r_{min}}^{\infty} dr\ \frac{r}{(r^2+z^2)^3}$$

(11a)

Where d_{min} and r_{min} accounts for the minimal distance between neighbor spins since the spins later can't be infinitesimally dense packed. Now assuming homogeneous distributed spins each caged in a sphere with radius of r_s, we make the following approach for a thick layer: $d_{min} \approx r_{min} \approx \frac{1}{\sqrt{2}} \cdot r_s$ and $\delta d \gg r_s$.

Spins in the center of the layer will have more neighbors to interact then spins directly at the surface. As an approximation we modify Equation (11a) by average over all possible positions:

$$\sum_{k\neq l}\frac{1}{r_{kl}^6}\approx\frac{\sigma}{\delta d-2d_{min}}\int_0^{\delta d-2d_{min}}dh\int_{-h}^{\delta d-2d_{min}-h}dz\int_0^{2\pi}d\phi\int_{r_{min}}^{\infty}dr\,\frac{r}{(r^2+z^2)^3} \tag{11b}$$

$$\sum_{k\neq l}\frac{1}{r_{kl}^6}\approx 2\pi\sigma\cdot\frac{\pi}{2\sqrt{2}r_s^3}\approx 7\cdot\frac{\sigma}{r_s^3} \tag{11c}$$

And the average spin-spin interaction rate of a spin bath in a thick layer can be approximated by:

$$R_{dip}^{layer}\approx 0.5157\cdot\mu_0\cdot\frac{g^2\cdot\mu^2}{\hbar}\cdot C_s\cdot\sqrt{\frac{\sigma}{r_s^3}} \tag{12}$$

Supplementary Discussion

NV spin population readout in NDs

The charge state of the NV centers can be influenced by various quantities, such as the local environments, surface treatment of the diamond, excitation wavelength of laser and etc.[10,11,12,13]. In particular, the charge state of NV centers in NDs is highly depending on the surface passivation due to their high surface to volume ratio[12]. In addition, their charge state can adjust over time without illumination, and also differs under laser illumination. As a consequence, the NV centers can change their charge state on the time scale of several µs to ms, depending on the used laser power and wavelength[13], which can be directly observed as an increase or decrease of the NV fluorescence after turning on the laser[10]. A typical single T_1 measurement containing no additional control sequence will contain that information, diminishing the measured spin contrast significantly. If one now applies a second measurement (control), i.e., by an additional microwave pulse (e.g., square or linear chirp pulse) before read out, the NV sublevel population is inverted and the spin contrast can be calculated. In other words, one can extract the pristine spin contrast by subtracting the normalized T_1 measurement from the control[14].

Simulation of NV spin sublevel flip induced by microwave excitation

We simulated the evolution of the spin-state using the NV spin Hamiltonian[15,16] by applying a linear polarized microwave excitation along the x-direction of the NV reference frame, while its z-

direction is along the NV rotational symmetry axis. We introduced the desired broadening as a Zeeman-like term, which would be introduced by a magnetic field aligned along the NV quantization axis. Both transitions are split by the “Zeeman-like term” around 16 MHz (see Supplementary Fig. 10). In this scenario we only have to consider the angular dependence of the microwave excitation field versus the NV quantitation axis. To simplify the simulated model, we also intentionally discard the Zero field parameter E that is accounting for strain in the NDs crystal. After averaging over all possible angular orientations of the microwave driving field, we extract the average probability to flip NV sublevel.

Inhomogeneous ODMR linewidth broadening in NDs

From Hahn-echo measurements on a well dispersed NDs sample we estimated an average T_2 time around ~1μs (data not shown) for single to few NVs in an individual diamond nanocrystal. This is a typical value for NDs[17,18,19]. The corresponding natural line width of one ODMR line would be around several hundreds of kHz, and therefore cannot be the origin of the observed line-broadening (Supplementary Fig. 10a). Another possible broadening by laser excitation could be ruled out by checking its laser power dependent behaviors (data not shown). Therefore, we attribute the observed inhomogeneous ODMR line broadening to the variations of strain among different diamond nanocrystals[20,21]. In addition, a weak residual static magnetic field, e.g., the earth magnetic field, may also partially account for such splitting and broadening.

Supplementary References

1. Schaefer, M., Hanik, N. & Kilbinger, A. F. M. ROMP Copolymers for Orthogonal Click Functionalizations. *Macromolecules* **45**, 6807–6818 (2012).

2. Moore, D. A. Selective Trialkylation of Cyclen with tert-Butyl Bromoacetate. *Org. Synth.* 10–14 (2008).

3. Wang, Y. *et al.* Reduction-Degradable Linear Cationic Polymers as Gene Carriers Prepared by Cu(I)-Catalyzed Azide−Alkyne Cycloaddition. *Biomacromolecules* **12**, 66–74 (2011).

4. Rudovský, J. *et al.* Synthesis of a bifunctional monophosphinic acid DOTA analogue ligand and its lanthanide(III) complexes. A gadolinium(III) complex endowed with an optimal water exchange rate for MRI applications. *Org. Biomol. Chem.* **3**, 112–117 (2005).

5. Gilchrist, T. L., Wasson, R. C., King, F. D. & Wootton, G. Intramolecular cycloaddition of azoalkenes derived from terminal alkenoic and alkynoic acids. *J. Chem. Soc. Perkin Trans. 1,* 2511–2516 (1987).

6. Yau, H. M., Croft, A. K. & Harper, J. B. Investigating the origin of entropy-derived rate accelerations in ionic liquids. *Faraday Discuss.* **154**, 365–371 (2011).

7. Jarmola, A., Acosta, V. M., Jensen, K., Chemerisov, S. & Budker, D. Temperature- and magnetic-field-dependent longitudinal spin relaxation in nitrogen-vacancy ensembles in diamond. *Phys. Rev. Lett.* **108**, 197601 (2012).

8. Tetienne, J. P. et al. Spin relaxometry of single nitrogen-vacancy defects in diamond nanocrystals for magnetic noise sensing. *Phys. Rev. B* **87**, 235436 (2013).

9. Caravan P., Ellison J. J., McMurry T. J. & Lauffer R. B. Gadolinium(III) Chelates as MRI Contrast Agents: Structure, Dynamics, and Applications. *Chem. Rev*. **99**, 2293 – 2352 (1999).

10. Gaebel T, et al. Photochromism in single nitrogen-vacancy defect in diamond. *Appl. Phys. B* **82**, 243-246 (2006).

11. Hauf M, et al. Chemical control of the charge state of nitrogen-vacancy centers in diamond. *Phys. Rev. B* **83**, 081304 (2011).

12. Petráková V, et al. Luminescence of nanodiamond driven by atomic functionalization: towards novel detection principles. *Adv. Funct. Mater*. **22**, 812-819 (2012).

13. Aslam N, Waldherr G, Neumann P, Jelezko F, Wrachtrup J. Photo-induced ionization dynamics of the nitrogen vacancy defect in diamond investigated by single-shot charge state detection. *New J. Phys.* **15**, 013064 (2013).

14. Häberle T, Schmid-Lorch D, Reinhard F, Wrachtrup J. Nanoscale nuclear magnetic imaging with chemical contrast. *Nat. Nanotechnol.* **10**, 125-128 (2015).

15. Michl J, et al. Perfect alignment and preferential orientation of nitrogen-vacancy centers during chemical vapor deposition diamond growth on (111) surfaces. *Appl. Phys. Lett.* **104**, 102407 (2014).

16. Alegre TPM, Santori C, Medeiros-Ribeiro G, Beausoleil RG. Polarization-selective excitation of nitrogen vacancy centers in diamond. *Phys. Rev. B* **76**, 165205 (2007).

17. Tisler J, et al. Fluorescence and spin properties of defects in single digit nanodiamonds. *ACS Nano* **3**, 1959-1965 (2009).

18. Laraoui A, Hodges JS, Meriles CA. Nitrogen-vacancy-assisted magnetometry of paramagnetic centers in an individual diamond nanocrystal. *Nano Lett.* **12**, 3477-3482 (2012).

19. Trusheim ME, et al. Scalable fabrication of high purity diamond nanocrystals with long-spin-coherence nitrogen vacancy centers. *Nano Lett.* **14**, 32-36 (2013).

20. Bradac C, et al. Observation and control of blinking nitrogen-vacancy centres in discrete nanodiamonds. *Nat. Nanotechnol.* **5**, 345-349 (2010).

21. McGuinness L, et al. Quantum measurement and orientation tracking of fluorescent nanodiamonds inside living cells. *Nat. Nanotechnol.* **6**, 358-363 (2011).
